# ^161^Tb-DOTATOC Production Using a Fully Automated Disposable Cassette System: A First Step Toward the Introduction of ^161^Tb into the Clinic

**DOI:** 10.2967/jnumed.122.265268

**Published:** 2023-07

**Authors:** Chiara Favaretto, Pascal V. Grundler, Zeynep Talip, Stefan Landolt, Lebogang Sepini, Ulli Köster, Cristina Müller, Roger Schibli, Susanne Geistlich, Nicholas P. van der Meulen

**Affiliations:** 1 Center for Radiopharmaceutical Sciences, ETH–Paul Scherrer Institute, Villigen-PSI, Switzerland;; 2 Department of Chemistry and Applied Biosciences, ETH, Zurich, Switzerland;; 3 Radiochemistry, South African Nuclear Energy Corp., Brits, South Africa;; 4 Institut Laue-Langevin, Grenoble, France; and; 5 Laboratory of Radiochemistry, Paul Scherrer Institute, Villigen-PSI, Switzerland

**Keywords:** ^161^Tb, specifications, DOTATOC, GMP compliant, automated

## Abstract

^161^Tb is an interesting radionuclide for application in the treatment of neuroendocrine neoplasms’ small metastases and single cancer cells because of its conversion and Auger-electron emission. Tb has coordination chemistry similar to that of Lu; therefore, like ^177^Lu, it can stably radiolabel DOTATOC, one of the leading peptides used for the treatment of neuroendocrine neoplasms. However, ^161^Tb is a recently developed radionuclide that has not yet been specified for clinical use. Therefore, the aim of the current work was to characterize and specify ^161^Tb and to develop a protocol for the synthesis and quality control of ^161^Tb-DOTATOC with a fully automated process conforming to good-manufacturing-practice guidelines, in view of its clinical use. **Methods:** ^161^Tb, produced by neutron irradiation of ^160^Gd in high-flux reactors followed by radiochemical separation from its target material, was characterized regarding its radionuclidic purity, chemical purity, endotoxin level, and radiochemical purity (RCP) in analogy to what is described in the European Pharmacopoeia for no-carrier-added ^177^Lu. In addition, ^161^Tb was introduced into a fully automated cassette-module synthesis to produce ^161^Tb-DOTATOC, as used for ^177^Lu-DOTATOC. The quality and stability of the produced radiopharmaceutical in terms of identity, RCP, and ethanol and endotoxin content were assessed by means of high-performance liquid chromatography, gas chromatography, and an endotoxin test, respectively. **Results:** ^161^Tb produced under the described conditions showed, as the no-carrier-added ^177^Lu, a pH of 1–2, radionuclidic purity and RCP of more than 99.9%, and an endotoxin level below the permitted range (175 IU/mL), indicating its appropriate quality for clinical use. In addition, an efficient and robust procedure for the automated production and quality control of ^161^Tb-DOTATOC with clinically applicable specifications and activity levels, that is, 1.0–7.4 GBq in 20 mL, was developed. The radiopharmaceutical’s quality control was also developed using chromatographic methods, which confirmed the product’s stability (RCP ≥ 95%) over 24 h. **Conclusion:** The current study demonstrated that ^161^Tb has appropriate features for clinical use. The developed synthesis protocol guarantees high yields and safe preparation of injectable ^161^Tb-DOTATOC. The investigated approach could be translated to other DOTA-derivatized peptides; thus, ^161^Tb could be successfully applied in clinical practice for radionuclide therapy.

## Visual Abstract

**Figure:**
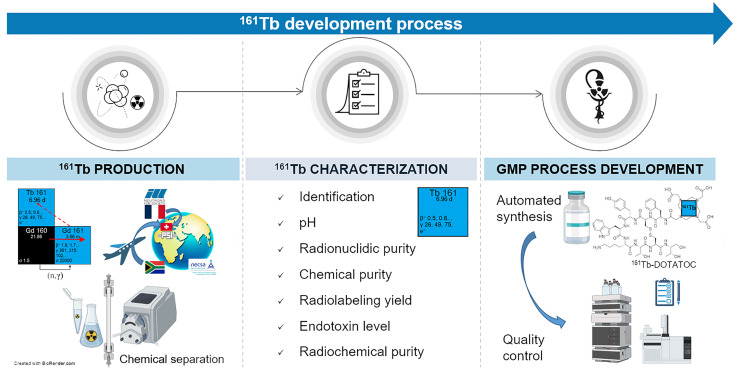

In recent years, peptide receptor radionuclide therapy (PRRT) has emerged as an option in metastatic or nonresectable neuroendocrine neoplasms (NENs) expressing high levels of somatostatin receptor (1*,*^2^). DOTATOC and DOTATATE are most commonly used for PRRT in NENs (3). In particular, in the neuroendocrine tumors therapy phase 3 randomized trial (4), ^177^Lu-DOTATATE treatment was confirmed as effective in tumor control with only minor side effects. It was approved by the European Medicines Agency in 2017 and the U.S. Food and Drug Administration in 2018 (Lutathera; Advanced Accelerator Applications) for the treatment of well-differentiated gastroenteropancreatic NENs (5*,*^6^). Despite its efficacy, studies have since shown partial remission of no more than 50%, with complete response of no more than 18% (4*,*^7^*,*^8^). The radionuclide ^161^Tb (Mean β^−^ energy, 154 keV [100%]; half-life, 6.96 d) (9*,*^10^) is proposed as a potential alternative to ^177^Lu (Mean β^−^ energy, 134 keV [100%]; half-life, 6.65 d) (11) because of their similar physical decay characteristics with regard to β^−^-particle emission, suitability for PRRT, and concomitant emission of photons, which can be used for SPECT imaging purposes (Table 1) (12*,*^13^). In addition, Tb has similar coordination chemistry to Lu (14); therefore, it can be stably coordinated with a DOTA chelator and respective tumor-targeting peptides, for example, DOTATOC or DOTATATE. ^161^Tb is regarded as superior to ^177^Lu because it coemits a substantial number of conversion and Auger electrons (∼12 e^−^, ∼37 keV per decay for ^161^Tb, ∼1 e^−^ and ∼1.0 keV per decay for ^177^Lu, respectively) (9*,*^11^*,*^13^), which would be more effective in the treatment of the smallest metastases, as well as single cancer cells (13*,*^15^–^19^). ^161^Tb production for radiopharmaceutical application was developed and is regularly performed at the Paul Scherrer Institute. It is produced by neutron irradiation of enriched ^160^Gd targets. The separated ^161^Tb product yields ^161^TbCl_3_ in a quality suitable for highly specific radiolabeling, which is useful for preclinical applications (13*,*^18^–^20^). At a clinical level, phantom studies were performed that demonstrated the feasibility of imaging ^161^Tb in high resolution when using low-energy, high-resolution collimators (21); a first-in-humans study was also conducted (22). The next step in the development of this radionuclide toward medical application is its introduction into drug manufacture under good manufacturing practice (GMP) to be able to demonstrate the higher efficacy of ^161^Tb-based radiopharmaceuticals than of ^177^Lu-labeled counterparts in clinical trials. An approach to demonstrate the sufficient quality of the ^161^TbCl_3_ solution for clinical purposes was to compare it with the specifications of commercially available no-carrier-added ^177^LuCl_3_, because the latter is approved for the preparation of several radiopharmaceuticals for clinical studies (4*,*^23^). One purpose of this study was to characterize ^161^Tb for clinical use. After this assessment, a protocol for the production of ^161^Tb-DOTATOC was developed, conforming to GMP principles, on an automated synthesis module.

**TABLE 1.** ^161^Tb Specifications Until Shelf-Life Expiration Date (9 Days After End of Separation)

Characteristic	Test	^161^TbCl_3_ specification
Appearance	Visual inspection	Clear and colorless solution
Identity	γ-spectrometry (keV)	74.6 ± 1, 87.9 ± 1, 103.1 ± 1, 106.1 ± 1, 292.4 ± 1, and 550.3 ± 1
pH	pH paper	1–2
Chemical purity	ICP-MS (μg/GBq)	Cu: <1.0
		Fe: <0.5
		Pb: <0.5
		Zn: <1.0
Sterility		Not required
Bacterial endotoxins	LAL test	<175 IU/mL (injectable dose)
Radionuclidic purity	γ-spectrometry	^160^Tb ≤ 0.1%
RCP	TLC	≥99.0% as ^161^TbCl_3_

ICP-MS = inductively coupled plasma–mass spectrometry; LAL = *Limulus* amebocyte lysate; TLC = thin-layer chromatography.

## MATERIALS AND METHODS

### ^161^Tb Production and Quality Control (QC)

^161^Tb was produced by neutron irradiation of enriched ^160^Gd_2_O_3_ targets (98.2% enrichment; Isoflex; Supplemental Table 1 [supplemental materials are available at http://jnm.snmjournals.org]), via the ^160^Gd(n,γ)^161^Gd→^161^Tb nuclear reaction at the South African Fundamental Atomic Research Installation (South African Nuclear Energy Corp.; ∼2·10^14^ n·cm^−1^·s^−1^ neutron flux) or the Institut Laue-Langevin’s High Flux Reactor (∼1·10^15^ n·cm^−1^·s^−1^ neutron flux). The quartz ampoule, containing the irradiated target material, was processed at the Paul Scherrer Institute for radiochemical separation of the produced ^161^Tb from the target material, as described previously (20). An approach to demonstrate the high quality of the ^161^TbCl_3_ product was to compare it with the specifications of commercially available no-carrier-added ^177^Lu, approved for the preparation of radiopharmaceuticals for clinical studies, described in the European Pharmacopoeia (Ph. Eur.) monograph (24).

#### Identity, pH, and Radionuclidic Purity

The identification and radionuclidic purity of ^161^Tb were examined by γ-spectrometry using a high-purity germanium detector (Canberra), in combination with the InterWinner software package (version 7.1; Itech Instruments; supplemental materials and Supplemental Fig. 1). The choice of γ-rays used for a reliable radionuclide identification accounted for possible interferences from other radionuclides that could create artifacts (supplemental materials). The pH of the ^161^TbCl_3_ solution was assessed by means of pH paper.

#### Chemical Purity

The chemical purity of the ^161^TbCl_3_ solution was assessed via inductively coupled plasma–mass spectrometry (Element HR; Thermo Fisher Scientific; supplemental materials). Four batches of ^161^Tb were analyzed after the radiochemical purification for contents of Cu, Fe, Pb, Zn, Gd, and Tb, as described in the Ph. Eur. monograph for ^177^Lu (24). The purity was also investigated in all 7 ^161^Tb productions performed during this study by means of apparent molar activity (AMA) determination through hand-operated labeling of the product to DOTATOC (supplemental materials), as described elsewhere (13*,*^20^).

#### Endotoxin Level

Because ^161^Tb is considered a radiopharmaceutical precursor, the bacterial endotoxin level needs to be tested. Pyrogens were tested according to Ph. Eur. standards using a chromogenic *Limulus* amebocyte lysate test (supplemental materials) (25). In the evaluation of the endotoxin level, international units per milliliter were used, considering that the unit used in the Ph. Eur. monograph is international units per volume, indicating the maximum volume to be used for the preparation of a single patient dose, which corresponds to 1 mL of ^161^Tb (maximum volume at the end of separation).

#### Radiochemical Purity (RCP)

The RCP of 3 ^161^Tb batches was assessed by thin-layer chromatography, as described in the Ph. Eur. monograph for ^177^Lu (supplemental materials) (24). Quantification of the thin-layer chromatography signals with the indicated software enabled the determination of the area of the peak of ^161^Tb in ionic form (R_f_ = 0.4–0.7), over the total activity.

### Automated Synthesis of ^161^Tb-DOTATOC

**FIGURE 1.**
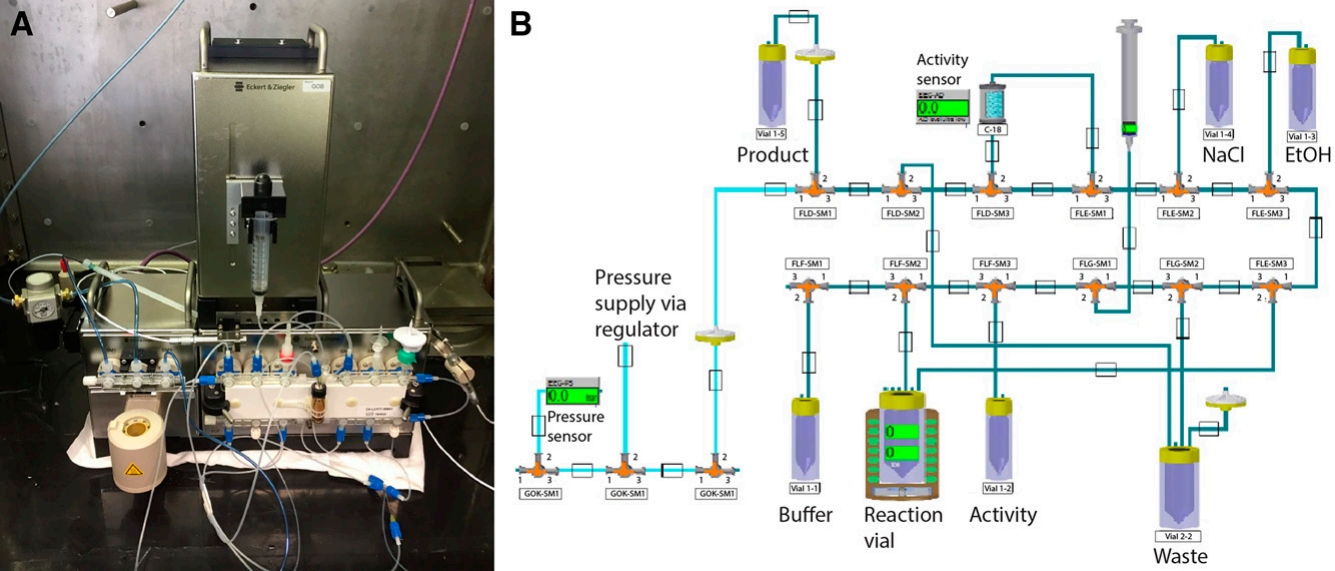
(A) Eckert & Ziegler Modular-Lab PharmTracer fully automated cassette synthesis system. (B) Graphical display of cassette components and synthesis process.

Ten syntheses of ^161^Tb-DOTATOC were performed using a Modular-Lab PharmTracer fully automated cassette synthesis system (Eckert & Ziegler; Fig. 1A), installed in a hot cell so that high activities of the radionuclide could be manipulated with no radiation dose exposure to the operator. For each synthesis, the sterile synthesis cassettes were prepared with the necessary reagents and attached to the Modular-Lab’s synthesis module (supplemental materials). A synthesis protocol was adequately adapted and optimized, and the production of ^161^Tb-DOTATOC was run by the Modular-Lab PharmTracer software (Modular-Lab SoftPLC; Eckert & Ziegler), which provided a graphical display of each step and the progress of the synthesis together with audit trail as required by GMP (Fig. 1B) (26*,*^27^). To ensure sterility, the final product was finally passed through a 0.22-μm filter (Medical Millex-GV Syringe Filter, Hydrophilic Polyvinylidene Fluoride, γ-sterilized; Merck Millipore). At the end of the synthesis, the final product activity and the residual radioactivity remaining in the principal components (activity vial, reaction vial, C18 cartridge, and waste) were measured (Isomed Dose Calibrator 2010) (28). Aliquots were withdrawn from the total volume for QC purposes.

### ^161^Tb-DOTATOC QC

#### High-Performance Liquid Chromatography (HPLC)

QC was performed to determine the RCP of ^161^Tb-DOTATOC, as well as the identification of the compound and possible impurities, by means of HPLC (Dionex P680 LGP Pump). First, the retention times of the product peaks in the radiochromatogram and UV chromatogram were assessed (Supplemental Figs. 2 and 3). Eventually, during the QC of ^161^Tb-DOTATOC, 20 μL of the synthesis product were analyzed by means of HPLC to estimate the RCP of ^161^Tb-DOTATOC from the HPLC radiodetector chromatogram and to identify the compound and possible impurities in the UV chromatogram.

Further evaluations of the method were performed to investigate the suitability and robustness of the QC. In particular, the linearity, limit of detection, and limit of quantification for both the radiodetector and the UV detector were assessed (Supplemental Fig. 4), together with the setup of a system suitability test (Supplemental Fig. 5).

#### Gas Chromatography

The ethanol content in the synthesis product was assessed by means of gas chromatography. The quantification of ethanol was based on the acetonitrile internal standard (supplemental materials). The linearity of the gas chromatography method was assessed by linear regression analysis for 6 ethanol concentrations between 1% and 15% (Supplemental Fig. 6).

#### Sterility and Endotoxin Level

Although the synthesis was not performed under GMP-compliant clean conditions, the pyrogenicity of the product and the integrity of the sterilization filter were evaluated to assess the suitability of the methods and to prove the acceptable quality of ^161^Tb-DOTATOC. Pyrogens were tested according to Ph. Eur. standards with a chromogenic *Limulus* amebocyte lysate test, as described earlier for the endotoxin test on the ^161^TbCl_3_ solution (25).

To ensure the integrity of the sterilization filter used at the end of the synthesis, a bubble point test was developed. The threshold value of the pressure depends on the matrix of the sterilized solution; therefore, it was assessed for the product in question (supplemental materials) (29). The bubble point test was then performed on the filter used during the synthesis of the batch tested for bacterial endotoxins.

#### Stability

The stability of the synthesis product, ^161^Tb-DOTATOC, was tested in both normal and stress conditions. To establish the stability of the radiolabeled peptide, all synthesized batches of ^161^Tb-DOTATOC were stored at room temperature and the RCP was assessed 3 and 24 h after synthesis. In addition, 1 batch of DOTATOC radiolabeled with high activity (8.77 GBq) was stored at room temperature for 96 h and the RCP was assessed by means of HPLC every 24 h with the method described earlier. After 96 h at room temperature, the product was incubated at 40 °C for an additional 72 h and HPLC analyses were conducted every 24 h.

## RESULTS

### ^161^Tb Production and QC

Multiple productions of ^161^Tb were analyzed, compared with the Ph. Eur. monograph for ^177^Lu, and found to comply with all requirements (Table 1).

#### Radionuclidic Purity

The content of the long-lived impurity ^160^Tb (half-life, 72.3 d) (30) was less than 0.005% of the total ^161^Tb activity at end of bombardment (EOB), as reported previously by our group (20). Three weeks after EOB, the level of ^160^Tb was still no more than 0.03% (Supplemental Fig. 1; Supplemental Table 2). Therefore, the specification for radionuclidic purity, which must apply until the end of shelf life, was prudently proposed for ^160^Tb at a level of 0.1%. With this level of impurity, the additional radiation dose to the patient was estimated to be about 0.4% (supplemental materials) (31).

#### Chemical Purity

The content of Cu, Fe, Pb, and Zn measured in the ^161^Tb samples was far below the limit for metal impurities reported for ^177^Lu (Supplemental Table 3). The Gd content was less than 0.4 ppm. Moreover, 6 of the 7 ^161^Tb productions were tested for AMA and successfully labeled DOTATOC at 100 MBq/nmol. Even so, the ^161^Tb production that allowed labeling of only the lower AMA was sufficiently pure to be used for ^161^Tb-DOTATOC synthesis (Table 2). Thanks to Tb quantification, the specific activity was also calculated and resulted in values greater than 3.5 GBq/μg.

**TABLE 2.** ^161^Tb Activity at EOS ^161^Tb Activity and Measured Before and at End of Synthesis for Each ^161^Tb-DOTATOC Production

Activity EOS (GBq)	Labeling yield at 100 MBq/nmol AMA (%)	Synthesis	Initial activity (GBq)	Bulk product activity (GBq)	Production yield (%)
13.4	99.9	1	1.29	1.12	86.8
		2	1.16	1.08	93.1
15.3	99.9	3	1.19	1.13	95.0
15.2	100	4	8.80	8.77	99.7
5.78	68.0	5	1.33	1.25	94.0
		6	2.87	2.79	97.2
11.7	99.9	7	9.26	8.92	96.3
11.8	99.7	8	9.17	9.08	99.0
14.3	100	9	9.39	9.26	98.6
		10	1.56	1.50	96.2

EOS = end of separation.

#### Endotoxin Level

The endotoxin content of the 3 batches tested was less than 1.20, 1.00, and 1.65 IU/mL, respectively, considerably lower than the requirement of less than 175 IU/mL.

#### RCP

In all productions tested, the peak of ^161^Tb in ionic form (R_f_ = 0.4–0.7) over the total activity (^161^Tb-diethylenetriaminepentaacetic acid, R_f_ = 0.9) was above 99.0%, thereby allowing the RCP of ^161^Tb to be established as greater than 99.0%.

### Automated Synthesis of ^161^Tb-DOTATOC

Ten preparations of ^161^Tb-DOTATOC were performed and form part of this work. For all syntheses, the ^161^Tb-labeled radioactivity at the shelf-life expiration date was in the range from 1 to 7.4 GBq ± 10%, which was the target to obtain a product comparable to a patient dose for ^177^Lu-DOTATOC (32). In particular, 5 syntheses produced ^161^Tb-DOTATOC with the minimum activity, 4 syntheses produced ^161^Tb-DOTATOC with the maximum activity, and 1 synthesis produced ^161^Tb-DOTATOC with intermediate activity (Table 2). The AMA was maintained at a constant of approximately 50 MBq/nmol, varying the peptide amount from 27 μg to a maximum of 200 μg (32). The duration of the process was approximately 45 min, and the total volume of the bulk product at the end of the synthesis was 24 mL, of which 4 mL were intended for QC and 20 mL were intended as final product. The final formulation was established in analogy to that of ^177^Lu-DOTATOC (Table 3).

**TABLE 3.** Composition of ^161^Tb-DOTATOC (bulk product)

Characteristic	Data
Range of ^161^Tb activity (shelf-life expiration date)	1–7.4 GBq
Volume	24 mL
DOTATOC and metal complexes	1 μg/37 MBq (maximum, 200 μg)
Calcium-DTPA	19.5 mg (0.1 mL, 195 mg/mL)
Ascorbic acid	250 mg (2.5 mL, 100 mg/mL)
Ethanol	1.25 mL (2.5 mL, ethanol 50%)
Saline	19 mL

DTPA = diethylenetriaminepentaacetic acid.

All syntheses produced ^161^Tb-DOTATOC within ±10% of stated parameters.

Residual radioactivity was detected in the activity vial, the reaction vial, the C18 cartridge, and the waste (Supplemental Table 4). Unlike the bulk product and initial activity measurements, which were performed with appropriate calibration for each container (28), the residual activity measurements were not comparable to one another, or to the initial activity, because of the distinct geometries of the samples. Nevertheless, the measurements indicated losses mainly in the waste or because of bound activity on the C18 cartridge. However, a total yield of more than 85% of ^161^Tb-DOTATOC, in reference to the initial ^161^Tb activity, was determined (Table 2).

### ^161^Tb-DOTATOC QC

The synthesis product complied with the established specifications (Table 4).

#### HPLC

**FIGURE 2.**
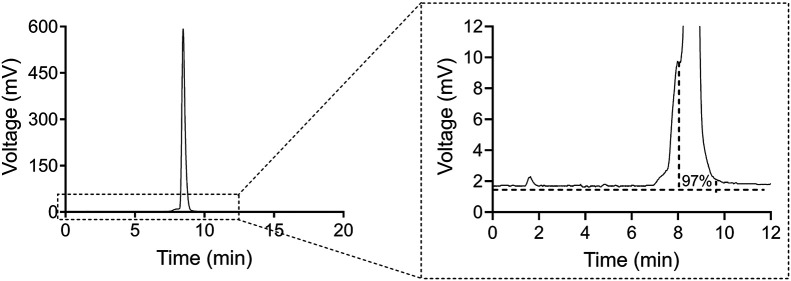
Representative HPLC radiochromatogram of high-activity batch of ^161^Tb-DOTATOC with RCP of 97.0% (1.6-min retention time would indicate ^161^Tb-diethylenetriaminepentaacetic acid, whereas 8.5 min indicates ^161^Tb-DOTATOC).

The results of the HPLC analysis of the synthesis products demonstrated that the RCP of ^161^Tb-DOTATOC was, on average, approximately 98% at the end of synthesis (*n* = 10; 98.2% ± 0.82; Supplemental Table 5), which meets the 95% specification level normally applied to these types of products (Fig. 2) (33*,*^34^).

**FIGURE 3.**
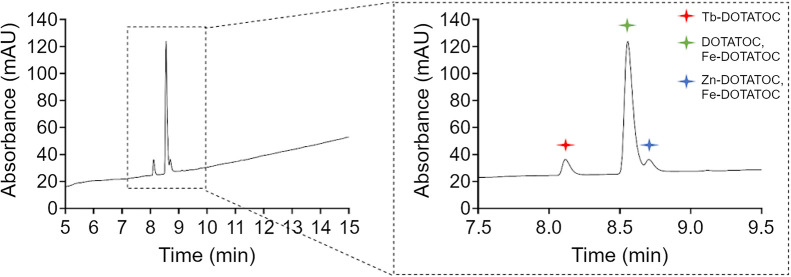
Representative HPLC UV chromatogram obtained during QC of synthesis product. Product peaks (DOTATOC, Tb-DOTATOC, Fe-DOTATOC, and Zn-DOTATOC) were identified, and no other peaks (impurities) were observed. milli Absorbance Unit.

The UV chromatograms showed low levels or complete nonappearance of impurities. The peaks of the unlabeled peptide (DOTATOC), together with those of Tb-DOTATOC, Fe-DOTATOC, and Zn-DOTATOC, established during the development of the HPLC method, were identified during the QC of the synthesis product and considered product peaks (Fig. 3).

#### Gas Chromatography

**FIGURE 4.**
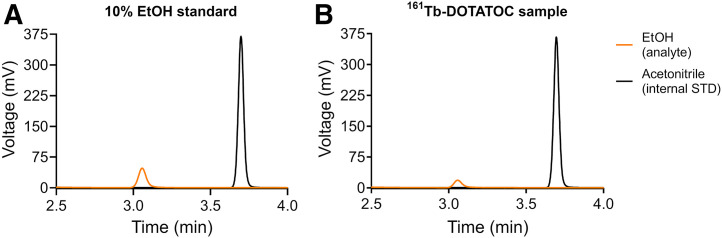
Representative gas chromatograms of 10% ethanol standard solution (A) and of sample of synthesis product (B). In both solutions, acetonitrile was added as internal standard (STD).

The ethanol content in the synthesis product was demonstrated to be less than 10%, which is the acceptable limit for radiopharmaceutical preparation for intravenous injection (35). In particular, the area of the ethanol peak in the analyzed sample of the synthesis product was normalized to the internal standard peak area and then compared with the normalized area of the ethanol peak in the 10% ethanol standard solution. In Figure 4, the ethanol peaks of the standard solution and the synthesis product are visually compared, with the lower ethanol content in the synthesis product displayed.

#### Sterility and Endotoxin Level

The synthesis resulted in a clear, colorless, particle-free product with a low level of bacterial endotoxins in the batch tested (<6.15 international units/mL). This endotoxin content was compliant with the specification for radiopharmaceutical products defined by the Ph. Eur. monograph (≤175 international units/20 mL) (25*,*^33^).

Although the sterility of the final product requires further validation, the integrity of the sterilization filter used at the end of the synthesis was successfully proven with a bubble point test. In particular, the bubble point was less than 2.75 bar, as specified for the product matrix (Supplemental Table 6).

#### Stability

**FIGURE 5.**
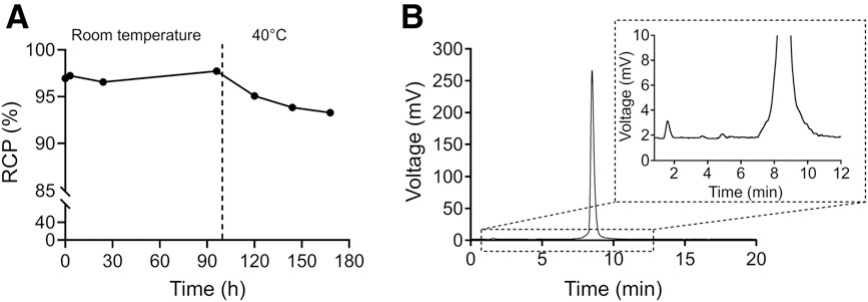
(A) ^161^Tb-DOTATOC (8.77-GBq batch) stability for 4 d at room temperature and additional 3 d at 40 °C. (B) HPLC radiochromatogram of ^161^Tb-DOTATOC after 48 h at 40 °C (RCP of 93.8%).

^161^Tb-DOTATOC presented RCP of more than 95% up to 24 h for all batches tested (*n* = 9; 97.6% ± 1.0 at 24 h; Supplemental Table 5). In addition, the RCP results of the batch selected for the stability test under stress conditions indicated that ^161^Tb-DOTATOC was highly stable at room temperature for up to 4 d (Fig. 5A). When the product was incubated at 40 °C, it demonstrated RCP of less than 95% over a period of 48 h, with increased released ^161^Tb and radiolysis (Fig. 5B).

**TABLE 4.** ^161^Tb-DOTATOC Specifications Until Shelf-Life Expiration Date

Characteristic	Test	Specification
Appearance	Visual inspection	Clear, colorless solution, without visible particles
pH	pH paper	4–8
Activity concentration	Dose calibrator	50–370 MBq/mL
RCP	HPLC	≥95%
^161^Tb-DOTATOC identity	HPLC	Peaks at defined RT in radio- and UV chromatograms
Impurities and degradation products	HPLC	Each impurity ≤ 100 μg/patient dose
Ethanol content	GC	<10%
Bacterial endotoxin level	LAL test	<175 IU/20 mL
Filter integrity (matrix-based)	bubble point test	Bubble point > 2.75 bar
Shelf life		24 h

RT = retention time; GC = gas chromatography.

## DISCUSSION

The aim of this work was to characterize ^161^Tb for clinical use, creating a list of parameters that can be the basis for future official specifications and monographic standards. The study compared ^177^Lu specifications with ^161^Tb features, showing that the latter comply with all requirements reported in the Ph. Eur. monograph for ^177^Lu (24). With regard to radionuclidic purity, long-lived ^160^Tb (half-life, 72.3 d) (30) was found as an impurity; it is coproduced by the ^159^Tb(n,γ)^160^Tb nuclear reaction, and the multistep reactions on the residual ^159^Tb, ^158^Gd, and ^157^Gd present as impurities in the target material. However, the content did not exceed 0.005% of the total ^161^Tb activity at EOB. Three weeks after EOB, the ingrowth of ^160^Tb was still no more than 0.03%. These results were observed in several productions after the irradiation of various amounts of target material with different neutron fluxes. According to previous studies by our group (20), the quality of ^161^Tb in terms of radiolabeling yield and AMA was comparable to that of commercially available ^177^Lu for 9 d after end of separation. This would prudently set the specifications to a shelf life of 9 d after end of separation and a radionuclidic purity of at least 99.9%. Because the latter would be true until 21 d after EOB, to comply with these specifications, the separation cannot be performed later than 12 d after EOB. However, these assumptions may no longer appear to be true if the target material has a different enrichment level from that used in this study; hence, more research is foreseen to examine this subject in greater detail. In addition, our earlier reports on the radiolabeling yield were supported by the results from this study with regard to ^161^Tb chemical purity and RCP. RCP tests showed that more than 99% of ^161^Tb was present in the chloride form, which is the expected chemical form after radiochemical separation, ensuring its direct use for radiolabeling. In terms of chemical impurities, the tested metals complied with the same requirement as stated for ^177^Lu. Moreover, only trace amounts of Gd were measured, implying the success of the radiochemical separation from the target material to provide ^161^Tb with a quality suitable for clinics. The specific activity resulted in more than 3.5 GBq/μg, which is comparable to what was achieved for commercially available no-carrier-added ^177^Lu (36). Because ^161^TbCl_3_ solution is not intended for direct administration to humans or for use in kit-type products at this stage of development, sterility does not need to be guaranteed (24). However, the product needs to be nonpyrogenic; therefore, pyrogens were tested according to Ph. Eur. standards with a chromogenic *Limulus* amebocyte lysate test (25), resulting in values below the recommended level.

Taking the preceding observations into consideration, one can see that ^161^Tb-labeled radiopharmaceuticals can easily be produced and made GMP-compliant for routine clinical use. The production, including synthesis and QC, of ^161^Tb-DOTATOC, the ^161^Tb equivalent of the somatostatin analogs currently used with ^177^Lu in clinical practice (37*,*^38^), was demonstrated. An Eckert & Ziegler cassette synthesis module, with a modified sequence, was applied to radiolabel DOTATOC with levels of ^161^Tb radioactivity suitable for a patient dose comparable to that used for ^177^Lu-DOTATOC, that is, 7.4 GBq in 20 mL (32). Ten productions were performed, yielding approximately 50 MBq/nmol AMA with up to 9.3 GBq of ^161^Tb-DOTATOC produced, which was enough to guarantee the required activity dose for a shelf life of 24 h. Precise activity measurement of ^161^Tb, which emits γ-radiation mainly with energies below 100 keV, becomes inaccurate with radionuclide dose calibrators used without appropriate calibration factors. An intensive investigation for the calibration of the dose calibrators with the geometries used in the manufacturing process was conducted in collaboration with the certified Institut de Radiophysique and is reported elsewhere (28). The 24 h-shelf life was established to reduce the activity use during the development work, but according to the stability studies, it could easily be extended for up to 4 d. The design of the synthesis system was based on disposable and sterilized flow paths that, together with the final filtration through a 0.22-mm filter, are expected to deliver a pharmaceutically adequate product (33*,*^34^). In addition, during the synthesis, ^161^Tb-DOTATOC was passed through a C18 cartridge to guarantee the removal of unreacted ^161^Tb. However, the final formulation contained diethylenetriaminepentaacetic acid to ensure the coordination of possible traces of released ^161^Tb, which is then quickly eliminated via renal excretion, preventing bone uptake of activity after the administration (39). The final product was then tested for RCP and ethanol and endotoxin content, which were determined to be in the acceptable ranges for pharmaceutical preparations. A tendency toward a lower RCP was observed in batches with high activity levels, which may be attributed to the greater activity concentration that caused the peptide to undergo more radiolysis. However, the RCP values for all products were far higher than the criterion for RCP of more than 95%. As a result of these assessments, further purification of the peptide was not required.

## CONCLUSION

In the present work, it was demonstrated that ^161^Tb shows quality standards comparable to those of ^177^Lu, implying its suitability for potential clinical use. In addition, an efficient and robust procedure for the automated production and QC of ^161^Tb-DOTATOC was developed using an automated cassette synthesis module and appropriate analytic techniques. The process described was evaluated in accordance with existing guidelines and quality standards. This will form the foundation of monographic standards for this nuclide and further attempts to introduce the use of Tb radioisotopes in clinical applications.

## DISCLOSURE

This work was financially supported by the Swiss National Science Foundation (200021_188495) and the NET Research Foundation Petersen Award. No other potential conflict of interest relevant to this article was reported.

## Notes

KEY POINTS **QUESTION:** Why is it important to develop a robust production of ^161^Tb and derived radiopharmaceuticals for the treatment of NENs? **PERTINENT FINDINGS:** The newly developed radionuclide ^161^Tb was characterized for clinical use, and fully automated synthesis of ^161^Tb-DOTATOC was achieved, showing positive results regarding the safety and quality of the product. **IMPLICATIONS FOR PATIENT CARE:** These findings would form the foundation of the introduction of ^161^Tb for clinical applications in the treatment of NENs, which could improve the outcome of the therapy because of its conversion and Auger electron emission targeting small metastases and single cancer cells.
